# Comparison of Sequence Reads Obtained from Three Next-Generation Sequencing Platforms

**DOI:** 10.1371/journal.pone.0019534

**Published:** 2011-05-17

**Authors:** Shingo Suzuki, Naoaki Ono, Chikara Furusawa, Bei-Wen Ying, Tetsuya Yomo

**Affiliations:** 1 Department of Bioinformatics Engineering, Graduate School of Information Science and Technology, Suita, Osaka, Japan; 2 Exploratory Research for Advanced Technology (ERATO), Japan Science and Technology Agency, Suita, Osaka, Japan; 3 Graduate School of Frontier Biosciences, Osaka University, Suita, Osaka, Japan; Charité-University Medicine Berlin, Germany

## Abstract

Next-generation sequencing technologies enable the rapid cost-effective production of sequence data. To evaluate the performance of these sequencing technologies, investigation of the quality of sequence reads obtained from these methods is important. In this study, we analyzed the quality of sequence reads and SNP detection performance using three commercially available next-generation sequencers, i.e., Roche Genome Sequencer FLX System (FLX), Illumina Genome Analyzer (GA), and Applied Biosystems SOLiD system (SOLiD). A common genomic DNA sample obtained from *Escherichia coli* strain DH1 was applied to these sequencers. The obtained sequence reads were aligned to the complete genome sequence of *E. coli* DH1, to evaluate the accuracy and sequence bias of these sequence methods. We found that the fraction of “junk” data, which could not be aligned to the reference genome, was largest in the data set of SOLiD, in which about half of reads could not be aligned. Among data sets after alignment to the reference, sequence accuracy was poorest in GA data sets, suggesting relatively low fidelity of the elongation reaction in the GA method. Furthermore, by aligning the sequence reads to the *E. coli* strain W3110, we screened sequence differences between two *E. coli* strains using data sets of three different next-generation platforms. The results revealed that the detected sequence differences were similar among these three methods, while the sequence coverage required for the detection was significantly small in the FLX data set. These results provided valuable information on the quality of short sequence reads and the performance of SNP detection in three next-generation sequencing platforms.

## Introduction

Three next-generation sequencing (NGS) technologies—Roche Genome Sequencer FLX System (FLX), Illumina Genome Analyzer (GA), and Applied Biosystems SOLiD system (SOLiD)—enable the rapid and cost-effective production of high-quality genome sequence data. These new technologies have demonstrated advantages over classical Sanger sequencing by capillary electrophoresis, such as the production of an enormous volume of sequence data inexpensively [1]. These new technologies have been successfully applied to whole-genome re-sequencing, *de novo* sequencing, transcriptomics, DNA methylation analysis, and metagenomics [2].

A significant feature of NGS is that it produces millions of short sequence reads for its analysis. The total amounts of data for each analysis are 450 Mbp for FLX, 18–35 Gbp for GA, and 30–50 Gbp for SOLiD, respectively, while the average lengths of each sequence read are 330 bp for FLA, 75–100 bp for GA, and currently 50 bp for SOLiD [1]. For the analysis of NGS data, it is necessary to assemble these millions of short sequence data to extract sequence features of DNA samples, such as detection of single nucleotide polymorphisms (SNPs) and *de novo* sequencing [3]. For such analysis, not only the total amounts of data, but also the quality of sequencing reads, such as error rate and systematic sequence bias in the obtained short reads, markedly impact the assembly results [4]. Thus, to evaluate the performance of NGS analysis, the quality of sequence reads should be investigated.

In this study, we evaluated the statistical nature of sequence reads and SNP detection performance using three commercially available NGS platforms, i.e., FLX, GA, and SOLiD. A common genomic DNA sample obtained from *E. coli* strain DH1 [5] was applied to three NGS platforms. The obtained short sequence reads were aligned to the complete genome sequence of DH1, which enabled us to evaluate the accuracy and systematic bias of sequence reads obtained from these three NGS platforms. Furthermore, we aligned the obtained sequence read to the complete genome of another *E. coli* strain, W3110 [6], to detect sequence differences between the genomes of two different *E. coli* strains. The results of these analyses revealed that, for example, about half of the sequence reads obtained by SOLiD could not be aligned to the reference genome, which suggested the fraction of “junk” data is significantly large in SOLiD. Among the data sets after alignment to the DH1 reference genome, the accuracy of sequence matching was significantly low in the data set of GA, suggesting a high error rate in GA data. The performance of SNP detection was similar among the three NGS platforms, while the coverage required for the SNP detection was significantly small in the data set of FLX, as expected from its relatively long sequence and high accuracy of FLX reads. These analyses provided valuable information on the quality of short sequence reads and the performance of SNP detection.

## Results and Discussion

### Determination of complete genome of *E. coli* DH1 ME856̀train using data of three next-generation sequencers

To investigate the quality of sequencing reads provided by NGS platforms, we analyzed whole-genome sequencing data of *E. coli* DH1 ME8569 strain. *E. coli* DH1 is a commonly used laboratory strain, which was constructed by serial genetic manipulations [5], and a finished version of the genome sequence of DH1 ATCC 33849 strain has recently been published (GenBank CP001637.1, submitted 2009). To determine the complete genome of the *E. coli* DH1 ME8569 strain used, we obtained sequence data using three different NGS platforms, i.e., FLX, GA, and SOLiD. Table 1 summarizes the volume of data from three NGS platforms. For SOLiD, we sequenced the DH1 ME8569 stain under different conditions using SOLiD including 25-base read data from a 3-kb mate pair library, two replicates of 50-base read data from fragment library, and two replicates of 50-base read data from a 3-kb mate pair library.

**Table 1:** Volume of data from three next-generation sequencing technologies.

Method	Read length	Number of reads	Total bases	Redundancy
FLX	260.7	475,819	124,042,803	26.8
GA	36	9,624,599	346,485,564	75
SOLiD M25	25	125,399,243	3,134,981,075	678.4
SOLiD M50	50	226,945,098	11,347,254,900	2455.4
SOLiD F50	50	100,015,475	5,000,773,750	1082.1

The read length of FLX was mean read length including adapters.

The SOLiD M25 was the data set of 25-base mate library.

The SOLiD M50 and F50 data sets were expressed as the sum of two replicates of 50-base mate pair and two replicates of 50-base fragment libraries, respectively.

To compare the quality of sequence reads obtained from three NGS platforms, first we determined the complete genome of DH1 ME8569 by an appropriate mix of these sequencing data, with Sanger sequencing for the uncovered genome sequences by three NGS methods (see Supplementary Text S1 for details). The obtained genome sequence of DH1 ME8569 had differences at 19 locations from that of DH1 ATCC33849 (GenBank CP001637.1, submitted 2009). The differences included three single-base substitutions, 8 short nucleotide insertions and deletions, and 8 structural differences. The complete information on the differences between two genomes of DH1 is presented in Supplementary Text S2.

### Comparison of accuracy of sequence reads

**Figure 1:**
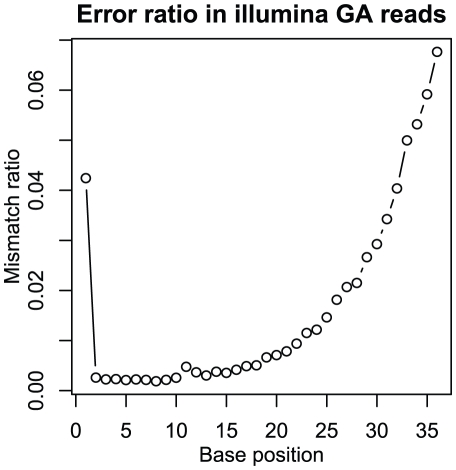
Error ratio in GA reads depending on the base position of the read. Ratio of mismatch between mapped reads and reference sequence to the total number of mapped reads was plotted against base position in the reads. The mismatch ratio increases along with the base position indicating decrease of accuracy of base calls.

Using the complete genome sequence of *E. coli* DH1 ME8569 as a reference, we evaluated the accuracy of sequence reads obtained from three NGS platforms by aligning these reads to the reference genome sequence. The same genomic DNA sample of *E. coli* strain DH1 ME8569 was sequenced using these three sequencing methods, and aligned to the reference. Although we had five data sets for ABI SOLiD (25-base mate pair, two replicates of 50-base fragments, and two replicates of 50-base mate pair libraries), we chose one data set (50-base mate pair library) for the comparison. For all analyses, we used the Bowtie [7] program and the same parameters to align these reads (see Materials and Methods for details), in which we allowed up to three mismatches in each alignment. On mapping of the reads of GA, mismatch ratio increased with the base position along the reads, which can be due to decrease of fluorescent signal intensities and quality of base calls. Since the Solexa base caller reports the quality of each base call as an estimated Quality Value (QV) similar to the phred score based on the image output [8], in order to filter out the affect of the errors due to these low quality base calls we trimmed the bases for which QV was lower than 0, and filtered out the trimmed reads with lengths shorter than 32 bases. Table 2 summarizes the results of these alignments of sequence reads. Here, “Ratio of mapped reads” represents the ratio of the number of reads mapped to DH1 ME8569 genome within three mismatches to the number of total reads, while “Accuracy” was defined as the ratio of the number of bases consistent with the reference sequence to the total number of mapped reads. The results showed that only around half of the reads were mapped to the reference in the data sets of SOLiD, indicating that half of the reads were not related to the genome sequence, i.e., “junk” data, which was the poorest quality among the three next-generation sequencers. Similar observations were reported previously [9]–[11]. There are several possible reasons for the many “junk” data derived from SOLiD. First, the adapters from sequencing chemistry would be read due to failure of the nick-translation step. Further, polyclonal beads containing multiple templates and fluorescence leakage of adjacent beads would result in “junk” data. In contrast, the FLX data set exhibited the highest ratio of mapped reads. As the average read length of the FLX data set was much larger than the others and the alignment was performed allowing up to three mismatches for all data sets, this result indicated that the quality of the FLX data set was much better than the other two methods. The accuracy of mapped sequence reads in the GA data set was significant lower than the other data sets. As shown in Figure 1, the inconsistent bases found in GA data sets were concentrated around the 3′ end of sequence reads. The concentration of inconsistencies at the 3′ end of sequence reads has been suggested to be due to accumulation of failures in incorporation of fluorescent dNTPs [8]. We could not find such position dependency of mismatch bases in mapped reads of the two other NGS methods.

**Table 2:** Comparison of mapping.

Method	Ratio of mapped reads	Accuracy per base
FLX	89.0	99.9
GA	63.7	96.7
SOLiD	47.3	99.8

Filtered data set of GA was shown.

### Comparison of uncovered regions

To gain a better understanding of systematic biases of each NGS platform, we analyzed the sequence composition of uncovered bases to which no reads were aligned. Table 3 shows the number of uncovered bases to which no reads were aligned in each data set. To identify the systematic biases in the uncovered bases, we removed the common 71334 uncovered bases from the uncovered bases by each platform. In the uncovered bases of Illumina GA, we found that the GC content was higher than that of the genome of *E. coli* DH1 ME8569 strain (50.8%). In Illumina GA, most of the uncovered bases were not concentrated in specific regions or did not span tens of base pairs, but one or a few uncovered bases were scattered over the whole genome. These observations suggested that G and C may increase the rate of sequence error in Illumina GA. We found no such tendency in the other two NGS methods.

**Table 3:** Comparison of uncovered regions.

Method	Uncovered bases (Uncommon)	GC contents
FLX	4,799	51.3
GA	58,367	56.1
SOLiD	27,986	50.4

The common 71334 uncovered bases not covered by any reads of the three methods were removed. Most were uncovered due to duplicated sequences, such as ribosomal RNA, insertion sequences, and highly preserved homologs.

### Comparison of detection of single-base substitutions

We evaluated detection of single-base substitutions using data sets obtained from three NGS platforms. The DH1 ME8569 genome determined here had 259 single-base substitutions with respect to the genome of the standard strain of *E. coli* W3110. To evaluate the performance of three NGS in detection of single-base substitutions, we mapped sequence reads obtained from the DH1 ME8569 genome to the W3110 genome as a reference, and screened substitutions using same method (see Materials and Methods for the details of the detection algorithm). Table 4 shows the number of detected substitutions using data sets of FLX, GA, and SOLiD. As shown in the table, the detection performances of single-base substitutions were similar in the data sets of FLX and SOLiD, while false positive and false negative rates were slightly higher in the data sets of GA. This difference may have been due to insufficient coverage of the GA data set for re-sequencing analysis, as discussed below. There were 15 substitutions that could not be detected by any of these methods; 12 of these 15 undetected substitutions were found in the regions of rRNA operons. The *E. coli* genome possesses 7 copies of the rRNA operon that show high degrees of identity to each other [6], and therefore reads in these regions were difficult to align correctly to the reference. The detected substitutions, the number of counted bases, and the quality values based on each method are listed in Supplementary Table S1.

**Table 4:** Detection of single-base substitutions.

Method	Coverage	False positive	True positive	False negative (Uncovered)
FLX	23.5	8	239	20 (14)
GA	16.8	46	223	36 (17)
SOLiD	609.3	18	243	16 (12)

**Figure 2:**
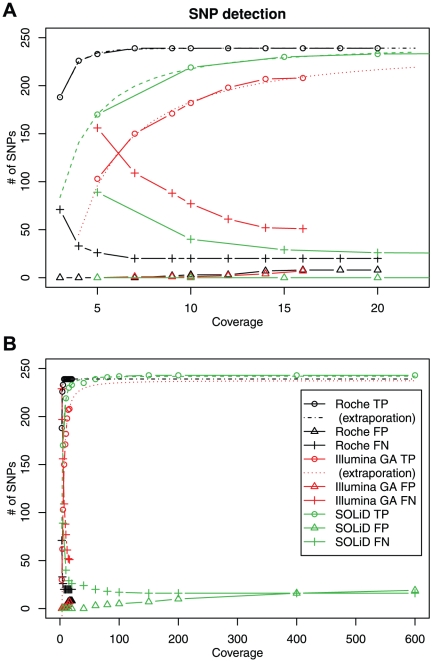
Numbers of true and false detection as a function of the mean coverage. (A) Magnification of the low coverage range. (B) Whole range. The circle, triangle and cross symbols indicate the number of True Positive (TP), False Negative (FN) and False Positive (FP), respectively. Black, red and green represent FLX, GA and SOLiD, respectively. The extrapolated lines for the saturation of TP using FLX and GA were added in (B).



Next, we evaluated how the true positive and false positive/negative rates depend on the number of sequence reads. In this analysis, we randomly resampled 1/32 to 1/2 (increasing by twofold) of total reads and using these resampled data sets with smaller size we screened for single-base substitutions using the identical algorithm as used in Table 4. Figure 2 shows true positive and false positive/negative rates as functions of mean coverage. As shown in the figures, the true positive and false negative rates were saturated around 10-fold coverage for FLX and 100- to 200-fold coverage for SOLiD, respectively. For the data set of GA, the true positive and false negative rates were not saturated, indicated that the coverage of GA data sets was insufficient for the detection of single-gene substitutions. The extrapolating curves of the true positive and false negative rates suggested that these were saturated around 50- to 100-fold coverage but the saturated false negative rate was slightly larger than for the other two methods. To evaluate how the number of TP depends on the coverage ***n*** for each method, we approximated it as a function of the coverage ***n*** by the following formulation,

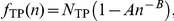
where 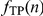 shows the number of detected TP and 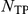 represents the total number of TP. Here, we assumed that the fraction of undetected substitution decreases as a power of the coverage ***n***, where A and B are fitting parameters.

## Methods

### Culture conditions, media, and genomic DNA preparation

*E. coli* DH1 [5] was obtained from the National BioResource Project at the National Institute of Genetics, Shizuoka, Japan. Glycerol stocked cells were inoculated into mM63 medium [12] and grown until OD_600_ = 0.5 with shaking at 130 rpm. The cell cultures were subsequently diluted to OD_600_ = 0.05 with fresh medium, and grown to stationary phase. Rifampicin (final concentration 300 (µg/mL) was subsequently added and culture was continued for a further 3 h to block initiation of DNA replication [13]. The cells were collected by centrifugation at 25°C at 16000×*g* for 5 min, and the pelleted cells were stored at −80°C prior to use. Genomic DNA was isolated and purified using an Aqua Pure Genomic DNA Isolation kit (Bio-Rad) in accordance with the manufacturer's instructions.

### Genome sequence analyses using Roche Genome Sequencer FLX System and Illumina Genome Analyzer

Sequence analyses were performed by Roche Diagnostic Japan for the Roche Genome Sequencer FLX System. Paired-end library preparation, sequencing, and base calling were performed according to the manufacturer's recommendations. The sequencing run was performed with an Illumina Genome Analyzer by Post Genome Institute. Two channels of a fragment flow cell were used. Fragment library preparation, sequencing, and base calling were performed according to the manufacturer's recommendations.

### Genome sequence by Applied Biosystems SOLiD system

The sequencing run with Applied Biosystems SOLiD 2 system was performed by Applied Biosystems Japan for the 25-base mate pair library. The other sequencing analyses for two replicates of 50-base fragment (two quarters of a slide) and two replicates of 50-base mate pair libraries (two quarters of a slide) were performed with an Applied Biosystems SOLiD 3 system. Library preparation, sequencing and base calling were performed according to the manufacturer's recommendations.

### Method for mutation detection

#### 1) Mapping to the assembled genome

We mapped all reads obtained by each three NGS method to the assembled DH1 genome sequence. To avoid issues related to the differences in mapping quality, we used the same mapping tool (Bowtie 0.12.3, http://bowtie-bio.sourceforge.net/index.shtml) for all data sets. All reads were mapped to the reference allowing up to three mismatches. Quality values of each sequence method were ignored in this mapping analysis. Adapter sequences were removed from the FLX reads. All reads were mapped regardless of mate pairs. In this mapping analysis, when a read could be mapped to multiple positions on the reference sequence, the one where the number of mismatches was the smallest was chosen; if there were multiple candidates for the least mismatch, the read was mapped to every position as if it was multiplied.

#### 2) Comparison of mutation detection

In this analysis, all reads of each NGS method were mapped to the genome sequence of *E. coli* W3110 as a reference by Bowtie allowing up to three mismatches. In this analysis, the reads that were mapped to multiple positions on the genome were excluded. The number of bases on the mapped reads was counted for each base position on the genome sequence. First, we estimated error rates of each sequencing methods by comparing the base types of the reference sequence and that of the mapped reads. Let 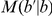 be the total number of bases *b*' appearing at all positions where the reference base is *b*. The read probability 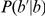 was defined as follows:
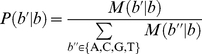
(1)


Next, for each base position *i,* we estimated the probability of base calling, i.e., what bases were in the read genome, from the counts of the mapped reads using this read probability. Let 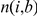 represent the number of bases *b* at position *i*. When the base counts a position 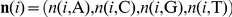 is given, the probability of the observation 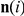 under the conditions that the base of the read genome is *b* is defined by multinomial distribution, as follows:
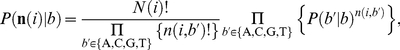
(2)where 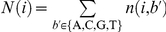.

Assuming that the prior probability distribution is uniform, the posterior probability of the read base is *b* under the given observation 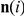 is given by Bayes' theorem as follows:
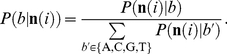
(3)


Among the four possible base types, we chose the one with the largest posterior probability as the consensus base at that position.

Finally, we filtered the bases where the consensus base was different from the reference base, and if the posterior probability of that consensus base was larger than 1×10^−8^, it was called a significant substitution.

### Data deposition

The complete genome sequence of *E. coli* DH1 ME856 strain has been submitted to GenBank under accession number AP012030.

## Supporting Information

Table S1 Information of the detected substitutions.
(DOC)

Text S1 Supplementary Methods.
(XLS)

Text S2 Supplementary Results.
(XLS)
