# Supplementary material for: Comparison of Sequence Reads Obtained from Three Next-Generation Sequencing Platforms
Source: PLoS One. 2011 May 17;6(5):e19534. doi: 10.1371/journal.pone.0019534 (PMC3096631; doi:10.1371/journal.pone.0019534)
Supplement: Supplementary file 1 [file pone.0019534.s001.doc]

## Sheet: SNP

Table S1 Information of the detected substitutions.
position in W3110	reference	detected in ME8569	FLX call	FLX QV	A	C	G	T	GA call	GA QV	A	C	G	T	SLD call	SLD QV	A	C	G	T
## FLX only
547829	G	FALSE	C	100	0	7	1	0	G	28	0	0	1	0	G	100	1	0	66	0
547830	C	FALSE	A	100	7	2	0	0	C	28	0	1	0	0	C	100	6	62	0	0
547831	A	FALSE	G	100	2	0	7	0	A	4	1	0	1	0	A	100	56	0	10	0
1434351	T	FALSE	C	100	0	4	0	0	T	29	0	0	0	1	T	100	0	1	0	67
3035697	C	FALSE	T	100	0	6	0	9	C	100	0	4	0	0	C	100	0	962	1	1
## GA only
264603	A	FALSE	A	100	31	0	0	0	C	100	0	5	0	0	A	100	459	1	0	0
369458	A	FALSE	A	100	24	0	0	0	C	100	1	4	0	0	A	100	409	0	0	0
403055	A	FALSE	A	100	14	0	0	0	C	91	0	3	0	0	A	100	303	0	0	0
423141	T	FALSE	T	100	0	0	0	24	G	91	0	0	3	0	T	100	0	0	0	692
590954	T	FALSE	T	100	0	0	0	20	G	100	0	0	5	2	T	100	0	2	1	711
736970	G	FALSE	G	100	0	0	22	0	T	100	0	0	1	4	G	100	0	0	198	0
790826	C	FALSE	C	100	0	21	0	0	A	100	5	2	0	0	C	100	0	825	2	1
853114	C	FALSE	C	100	0	22	0	0	G	100	0	1	4	0	C	100	1	711	0	1
1033527	A	FALSE	A	100	29	0	0	0	C	91	0	3	0	0	A	100	322	0	0	1
1153842	T	FALSE	T	100	0	0	0	20	G	91	0	0	3	0	T	100	3	2	2	742
1201445	C	FALSE	A	30	1	0	0	0	A	100	8	4	0	0	C	100	39	133	28	0
1202813	C	FALSE	C	100	0	22	0	0	A	100	6	3	0	0	C	100	0	334	0	0
1288574	A	FALSE	A	100	13	0	0	0	C	100	1	5	0	0	A	100	308	11	0	4
1434003	G	FALSE	G	100	0	0	25	0	T	100	0	0	1	4	G	100	0	0	394	3
1592866	C	FALSE	C	100	0	12	0	0	A	100	6	3	0	0	C	100	0	772	0	0
1651881	T	FALSE	T	100	0	0	0	25	G	91	0	0	3	0	T	100	0	0	0	77
1717038	T	FALSE	T	100	0	0	0	19	G	100	0	0	4	1	T	100	0	0	0	484
1847236	A	FALSE	A	100	11	0	0	0	C	100	1	4	0	0	A	100	257	0	0	0
2074635	A	FALSE	A	100	21	0	0	0	C	91	0	3	0	0	A	100	555	0	1	1
2294449	A	FALSE	A	100	10	0	0	0	C	100	3	7	0	0	A	100	137	0	0	0
2829155	T	FALSE	T	100	0	0	0	16	G	100	0	0	4	1	T	100	0	0	0	468
3066643	T	FALSE	T	100	0	0	0	15	G	100	0	0	5	2	T	100	0	0	1	547
3074775	G	FALSE	G	100	0	0	19	0	T	96	0	0	0	3	G	100	0	0	34	0
3582508	T	FALSE	T	100	0	0	0	19	G	91	0	0	3	0	T	100	0	1	0	296
3609157	A	FALSE	A	100	28	0	0	0	C	91	0	3	0	0	A	100	44	0	0	0
3622072	T	FALSE	T	100	0	0	0	27	G	91	0	0	3	0	T	100	0	0	0	827
3698241	G	FALSE	G	60	0	0	2	0	A	100	6	0	2	0	G	100	22	0	52	0
3698244	T	FALSE	T	96	0	0	0	3	G	100	0	0	4	1	T	100	0	0	0	112
3771203	A	FALSE	A	100	13	0	0	0	C	100	2	5	0	0	A	100	489	2	1	4
3925945	A	FALSE	A	100	23	0	0	0	C	100	2	5	0	0	A	100	502	0	0	0
3936239	T	FALSE	T	100	0	0	0	25	G	91	0	0	3	0	T	100	0	2	0	332
4034498	A	FALSE	A	100	20	0	0	0	C	100	1	4	0	0	A	100	656	2	1	0
4228081	A	FALSE	A	100	28	0	0	0	C	100	1	4	0	0	A	100	64	0	0	0
4351627	A	FALSE	A	100	14	0	0	0	C	100	1	4	0	0	A	100	559	0	0	6
4558085	T	FALSE	T	63	0	0	0	2	G	91	0	0	3	0	T	100	1	0	0	218
4621618	T	FALSE	T	100	0	0	0	20	G	100	0	0	5	2	T	100	0	0	1	566
4643675	A	FALSE	A	100	4	0	0	0	G	100	2	0	6	0	A	100	385	0	0	0
## SOLiD only
2320795	G	FALSE	C	28	0	1	0	0	C	3	0	1	1	0	C	100	0	27	0	0
2412225	C	FALSE	T	29	0	0	0	1	T	71	1	0	0	3	T	100	0	0	1	8
2813115	A	FALSE	T	29	0	0	0	1	A	3	2	0	0	2	T	100	2	1	0	30
2813117	C	TRUE	A	30	1	0	0	0	A	3	2	0	0	2	T	100	3	2	1	20
3596058	C	TRUE	T	66	0	1	0	3	C	28	0	2	0	1	T	100	0	88	0	344
3698240	G	FALSE	A	64	2	0	0	0	G	85	2	0	5	1	A	100	39	0	33	0
3744263	A	FALSE	T	63	0	0	0	2	T	63	0	0	0	2	T	100	6	0	0	15
## FLX and GA
4311557	C	FALSE	T	96	0	0	0	3	T	96	0	0	0	3	T	29	0	0	0	1
## FLX and SOLiD
10696	C	TRUE	T	100	0	0	0	18	T	63	0	0	0	2	T	100	4	5	3	362
283759	T	TRUE	G	100	0	0	13	0	G	60	0	0	2	0	G	100	0	1	92	1
1386366	A	TRUE	G	100	0	0	19	0	G	28	0	0	1	0	G	100	2	0	105	0
3119442	G	TRUE	A	100	15	0	0	0	A	30	1	0	0	0	A	100	127	7	9	1
3387896	T	TRUE	G	100	0	0	26	0	G	72	0	0	3	1	G	100	1	1	331	5
4051628	C	TRUE	T	100	0	0	0	77	T	34	0	1	0	2	T	100	1	0	0	18
4080795	G	TRUE	A	100	20	0	0	0	A	33	2	1	1	0	A	100	270	1	12	1
4228073	C	TRUE	T	100	0	0	0	28	T	29	0	0	0	1	T	100	0	0	1	24
4301678	C	TRUE	T	100	0	0	0	59	T	29	0	0	0	1	T	100	0	16	0	169
4304550	C	TRUE	T	100	0	0	0	15	T	63	0	0	0	2	T	100	2	8	0	416
4327283	C	TRUE	T	100	0	0	0	33	T	63	0	0	0	2	T	100	0	7	0	232
4330515	C	TRUE	T	100	0	0	0	19	T	29	0	0	0	1	T	100	0	0	0	12
4506180	C	TRUE	T	100	0	0	0	20	T	63	0	0	0	2	T	100	1	7	2	350
4558351	C	TRUE	T	100	0	0	0	7	T	63	0	0	0	2	T	100	0	3	2	142
4642738	C	TRUE	T	100	0	0	0	22	T	75	0	0	1	3	T	100	0	0	0	19
## GA and SOLiD
4310363	G	FALSE	C	28	0	1	0	0	C	100	0	5	0	0	C	100	6	16	2	0
4310364	G	FALSE	A	30	1	0	0	0	A	97	3	0	0	0	A	100	9	1	0	0
## All
1903	G	TRUE	A	100	21	0	0	0	A	100	16	1	0	1	A	100	657	0	9	0
23501	C	TRUE	T	100	0	0	0	19	T	100	0	0	0	7	T	100	0	14	3	245
31683	C	TRUE	T	100	0	0	0	18	T	100	0	0	0	15	T	100	3	19	2	206
42108	G	TRUE	A	100	11	0	0	0	A	100	29	0	0	0	A	100	261	0	14	0
59968	C	TRUE	T	100	0	0	0	18	T	100	0	0	0	23	T	100	4	15	2	343
76249	G	TRUE	A	100	27	0	0	0	A	100	11	3	0	0	A	100	428	0	16	2
88571	G	TRUE	A	100	17	0	0	0	A	100	7	1	0	0	A	100	550	0	5	0
113695	G	TRUE	A	100	28	0	0	0	A	100	28	1	0	0	A	100	854	2	5	5
156068	A	TRUE	G	100	0	0	15	0	G	100	0	0	33	0	G	100	2	0	271	0
157585	C	TRUE	T	100	0	0	0	19	T	100	0	0	1	9	T	100	3	2	0	405
276123	G	TRUE	A	100	14	0	0	0	A	100	8	0	1	1	A	100	310	0	5	2
280515	G	TRUE	A	100	27	0	1	0	A	97	3	0	0	0	A	100	215	3	15	2
282562	G	TRUE	A	100	22	0	0	0	A	100	6	0	0	0	A	100	328	0	5	2
285793	G	TRUE	A	100	23	0	0	0	A	100	5	0	0	0	A	100	233	5	5	0
289399	G	TRUE	A	100	14	0	0	0	A	100	10	0	0	0	A	100	410	2	1	0
291657	G	TRUE	A	100	18	0	0	0	A	100	35	0	0	1	A	100	385	0	4	1
297665	G	TRUE	A	100	16	0	0	0	A	100	7	2	0	0	A	100	120	3	12	0
302765	G	TRUE	A	100	10	0	0	0	A	100	38	2	0	1	A	100	272	3	12	1
305247	G	TRUE	A	100	25	0	0	0	A	100	15	0	0	0	A	100	831	1	11	0
310941	G	TRUE	A	100	18	0	0	0	A	100	25	0	0	0	A	100	229	0	8	0
312089	G	TRUE	A	100	15	0	0	0	A	100	25	0	0	0	A	100	567	2	4	3
312767	G	TRUE	A	100	23	0	0	0	A	100	23	0	0	0	A	100	733	1	8	1
317012	C	TRUE	T	100	0	0	0	16	T	100	0	0	1	11	T	100	3	2	0	724
324618	G	TRUE	A	100	21	0	0	0	A	100	42	4	1	0	A	100	453	21	32	7
327223	G	TRUE	A	100	21	0	0	0	A	100	16	0	1	1	A	100	370	0	2	1
331103	G	TRUE	A	100	12	0	0	0	A	100	36	0	0	0	A	100	564	1	4	0
337880	G	TRUE	A	100	13	0	0	0	A	100	4	0	1	0	A	100	163	0	15	0
339426	G	TRUE	A	100	25	0	0	0	A	100	27	0	0	0	A	100	102	0	6	0
339472	G	TRUE	A	100	18	0	0	0	A	100	16	0	0	0	A	100	425	2	6	1
342747	G	TRUE	A	100	26	0	0	0	A	100	18	2	1	0	A	100	328	1	2	0
348237	G	TRUE	A	100	18	0	0	0	A	100	6	0	0	0	A	100	492	1	6	0
357708	G	TRUE	A	100	17	0	0	0	A	97	3	0	0	0	A	100	347	10	40	31
358186	G	TRUE	A	100	30	0	0	0	A	100	13	2	0	0	A	100	720	0	3	0
361954	G	TRUE	A	100	24	1	0	0	A	100	4	1	0	0	A	100	394	4	0	0
364531	G	TRUE	A	100	25	0	0	0	A	100	11	0	1	0	A	100	307	0	4	2
367036	G	TRUE	A	100	19	0	0	0	A	100	7	2	0	0	A	100	290	2	9	0
389311	G	TRUE	A	100	15	0	0	0	A	100	19	0	0	1	A	100	374	3	14	6
465372	G	TRUE	A	100	21	0	0	0	A	100	17	0	0	0	A	100	506	0	8	1
468495	G	TRUE	A	100	24	0	0	0	A	100	6	1	0	0	A	100	479	1	3	0
479519	G	TRUE	A	100	20	0	0	0	A	100	28	1	0	0	A	100	36	0	10	0
479520	G	TRUE	A	100	20	0	0	0	A	100	27	0	0	0	A	100	36	1	7	1
501456	G	TRUE	A	100	21	0	0	0	A	100	13	0	1	0	A	100	382	0	7	0
547694	A	TRUE	G	100	0	0	12	0	G	100	0	0	16	0	G	100	4	0	414	0
662691	C	TRUE	T	100	0	0	0	18	T	100	0	0	1	14	T	100	6	11	0	403
785099	G	TRUE	A	100	21	0	0	0	A	100	12	0	0	0	A	100	580	1	2	1
881953	A	TRUE	T	100	0	1	0	14	T	100	0	0	0	21	T	100	3	1	0	536
1148773	C	TRUE	T	100	0	0	0	31	T	100	0	0	1	8	T	100	0	3	1	576
1158490	G	TRUE	A	100	15	0	0	0	A	100	6	0	0	0	A	100	678	2	1	1
1171413	A	TRUE	G	100	0	0	13	0	G	100	0	0	4	0	G	100	1	2	439	0
1191557	A	TRUE	G	100	0	0	20	0	G	100	0	1	25	5	G	100	59	13	484	8
1193221	C	TRUE	T	100	0	0	0	26	T	100	0	0	0	16	T	100	1	4	3	585
1284204	C	TRUE	T	100	0	0	0	24	T	100	0	0	1	15	T	100	0	6	1	858
1292246	G	TRUE	A	100	19	0	0	0	A	100	5	0	0	0	A	100	308	2	7	2
1299073	G	TRUE	A	100	14	0	0	0	A	100	9	1	0	0	A	100	746	3	21	2
1303706	A	TRUE	T	100	0	0	0	34	T	100	0	1	0	14	T	100	1	3	0	588
1308450	T	TRUE	G	100	0	0	17	0	G	100	0	0	6	1	G	100	0	0	290	0
1324473	G	TRUE	A	100	18	0	0	0	A	100	21	0	0	0	A	100	207	19	18	9
1328525	C	TRUE	G	100	0	0	17	0	G	100	0	0	15	0	G	100	0	4	251	1
1360573	T	TRUE	C	100	0	22	0	0	C	100	2	6	0	0	C	100	0	468	1	3
1403607	G	TRUE	A	100	18	0	0	0	A	100	31	0	0	1	A	100	719	10	21	3
1455706	G	TRUE	A	100	22	0	0	0	A	100	11	0	0	0	A	100	731	1	22	0
1599078	T	TRUE	C	100	0	15	0	0	C	100	1	20	0	0	C	100	6	231	0	4
1645393	A	TRUE	T	100	0	0	0	9	T	100	0	0	1	7	T	100	5	0	0	420
1777814	G	TRUE	A	100	17	0	0	0	A	100	12	0	0	0	A	100	504	2	3	1
1896553	T	TRUE	C	100	0	27	0	0	C	100	0	13	0	0	C	100	1	503	2	4
1956724	G	TRUE	A	100	20	0	0	0	A	100	6	0	0	0	A	100	314	1	33	10
1996774	A	TRUE	G	100	0	0	17	0	G	100	0	0	19	0	G	100	2	0	340	3
2034560	A	TRUE	G	100	0	0	21	0	G	100	0	0	22	1	G	100	5	2	496	3
2042570	C	TRUE	A	100	25	0	0	0	A	100	11	0	0	0	A	100	582	12	3	4
2043548	G	TRUE	A	100	18	0	0	0	A	100	21	0	0	0	A	100	721	2	11	3
2048643	G	TRUE	T	100	0	0	0	29	T	100	0	0	1	22	T	100	0	0	11	971
2087456	G	TRUE	A	100	24	0	0	0	A	100	8	2	0	0	A	100	487	1	1	4
2088771	G	TRUE	A	100	30	0	1	0	A	100	17	0	0	0	A	100	671	1	14	2
2106028	G	TRUE	A	100	15	0	0	0	A	100	20	0	0	0	A	100	377	1	13	5
2125045	G	TRUE	A	100	31	0	0	0	A	100	20	1	0	1	A	100	365	4	10	0
2136635	G	TRUE	A	100	26	0	0	0	A	100	15	0	0	0	A	100	255	4	11	2
2139680	G	TRUE	A	100	13	0	0	0	A	100	35	0	0	0	A	100	120	0	2	2
2175583	C	TRUE	T	100	0	1	0	20	T	100	0	0	0	15	T	100	4	15	1	378
2191421	T	TRUE	C	100	0	15	0	0	C	100	0	24	0	0	C	100	1	625	2	2
2333879	A	TRUE	G	100	0	0	29	0	G	100	0	0	7	1	G	100	12	19	481	26
2343832	C	TRUE	T	100	0	0	0	21	T	100	0	0	0	6	T	100	3	24	24	612
2415865	G	TRUE	A	100	24	0	0	0	A	100	5	0	0	0	A	100	412	4	10	1
2490273	C	TRUE	T	100	0	0	0	12	T	100	0	0	0	10	T	100	2	34	1	743
2516055	C	TRUE	T	100	0	0	0	38	T	100	0	0	0	6	T	100	2	5	0	379
2585768	C	TRUE	T	100	0	0	0	18	T	100	0	0	0	6	T	100	0	25	2	282
2731648	G	TRUE	A	100	20	0	0	0	A	100	13	0	0	0	A	100	459	4	36	2
2802789	C	TRUE	T	100	0	0	0	24	T	100	0	0	0	40	T	100	1	24	0	480
2815853	G	TRUE	A	100	13	0	0	0	A	100	14	0	0	0	A	100	433	0	1	2
2821944	C	TRUE	T	100	0	0	0	26	T	100	0	0	1	11	T	100	0	6	1	472
2865825	A	TRUE	G	100	0	0	26	0	G	100	0	0	11	0	G	100	14	9	270	3
2866110	T	TRUE	A	100	14	0	0	0	A	100	23	1	0	0	A	100	323	0	0	4
2866111	A	TRUE	G	100	0	0	14	0	G	100	0	0	23	2	G	100	1	0	322	0
2869809	T	TRUE	G	100	0	0	21	0	G	100	0	1	11	1	G	100	1	1	330	6
2925199	C	TRUE	T	100	0	0	0	23	T	100	0	0	1	10	T	100	0	16	0	48
2925200	C	TRUE	T	100	0	0	0	24	T	100	0	0	1	12	T	100	0	9	0	50
2925428	C	TRUE	A	100	17	0	0	0	A	100	16	0	0	0	A	100	436	1	1	2
3027202	G	TRUE	A	100	25	0	0	0	A	100	9	0	0	0	A	100	417	0	4	4
3049128	G	TRUE	A	100	9	0	0	0	A	100	4	1	0	0	A	100	201	0	3	1
3060572	C	TRUE	T	100	0	0	0	16	T	100	0	1	0	18	T	100	6	10	1	437
3064105	G	TRUE	A	100	28	0	0	0	A	100	10	1	0	0	A	100	186	2	5	0
3064517	G	TRUE	A	100	25	0	0	0	A	100	6	0	0	0	A	100	452	7	18	4
3087916	T	TRUE	C	100	0	19	0	0	C	100	0	4	0	0	C	100	0	701	0	3
3089624	G	TRUE	A	100	18	0	0	0	A	100	15	0	0	0	A	100	524	8	6	1
3103543	G	TRUE	A	100	23	0	0	0	A	100	15	0	0	0	A	100	456	2	3	0
3119689	T	TRUE	C	100	0	21	0	0	C	100	0	8	0	0	C	100	28	487	1	3
3126637	G	TRUE	A	100	21	0	0	0	A	100	16	3	0	0	A	100	442	2	9	3
3128817	G	TRUE	A	100	19	0	0	0	A	100	9	0	0	0	A	100	88	0	2	7
3130733	G	TRUE	A	100	13	0	0	0	A	100	9	3	0	0	A	100	645	0	8	0
3131831	G	TRUE	A	100	17	0	0	0	A	100	15	1	0	0	A	100	412	7	4	0
3132919	G	TRUE	A	100	18	0	0	0	A	100	15	1	0	0	A	100	474	0	4	1
3144600	G	TRUE	A	100	14	0	0	0	A	100	27	0	0	0	A	100	808	0	5	1
3148972	G	TRUE	A	100	27	0	0	0	A	100	13	1	0	0	A	100	407	0	25	5
3160872	G	TRUE	A	100	25	0	0	0	A	100	12	1	0	0	A	100	701	1	2	4
3175281	G	TRUE	A	100	13	0	0	0	A	100	14	0	0	0	A	100	220	1	5	2
3178039	G	TRUE	A	100	25	0	0	0	A	100	28	0	0	0	A	100	376	16	17	13
3192007	G	TRUE	A	100	14	0	0	0	A	100	9	0	0	0	A	100	545	2	7	1
3203273	G	TRUE	A	100	36	0	0	0	A	100	14	1	0	0	A	100	467	0	3	0
3217784	G	TRUE	A	100	16	0	0	0	A	100	7	1	0	0	A	100	432	2	7	5
3223551	C	TRUE	T	100	0	0	0	28	T	100	0	0	1	8	T	100	13	20	3	239
3225995	C	TRUE	T	100	0	0	0	11	T	100	0	0	1	8	T	100	2	3	1	473
3239079	C	TRUE	T	100	0	0	0	20	T	100	0	0	0	8	T	100	10	15	52	660
3254813	C	TRUE	T	100	0	0	0	20	T	100	0	0	0	13	T	100	5	5	2	671
3257939	G	TRUE	A	100	7	0	0	0	A	100	15	0	0	0	A	100	799	2	6	1
3270352	C	TRUE	T	100	0	0	0	18	T	96	0	0	0	3	T	100	0	1	0	278
3275757	A	TRUE	T	100	0	0	0	24	T	100	0	0	1	10	T	100	4	20	34	616
3292167	C	TRUE	T	100	0	1	0	26	T	100	0	0	0	16	T	100	0	7	2	546
3304502	T	TRUE	C	100	0	15	0	0	C	100	2	6	0	0	C	100	8	611	5	6
3329598	G	TRUE	T	100	0	0	0	12	T	100	0	0	0	21	T	100	0	0	2	557
3442861	G	TRUE	A	100	20	0	0	0	A	100	5	1	0	0	A	100	580	0	2	3
3443504	G	TRUE	A	100	25	0	0	0	A	100	6	0	0	0	A	100	119	0	9	0
3450904	G	TRUE	A	100	18	0	0	0	A	100	29	2	0	0	A	100	610	3	6	2
3457680	G	TRUE	A	100	20	0	0	0	A	100	10	0	1	0	A	100	506	0	0	0
3457798	G	TRUE	A	100	22	0	0	0	A	100	21	0	0	0	A	100	731	1	7	0
3460854	G	TRUE	A	100	18	0	0	0	A	100	28	1	1	1	A	100	219	6	7	1
3476524	C	TRUE	T	100	0	0	0	26	T	100	0	0	0	26	T	100	5	20	2	708
3497383	C	TRUE	T	100	0	0	0	20	T	100	0	0	1	11	T	100	0	3	1	770
3551749	C	TRUE	A	100	16	0	0	0	A	100	21	0	0	0	A	100	587	9	3	13
3680678	C	TRUE	T	100	0	0	0	17	T	100	0	0	2	9	T	100	1	14	3	241
3686582	G	TRUE	A	100	17	0	0	0	A	100	9	0	0	1	A	100	424	0	29	1
3698684	C	TRUE	T	100	0	0	0	17	T	100	0	0	0	13	T	100	2	7	6	779
3698772	C	TRUE	T	100	0	0	0	22	T	100	0	0	0	17	T	100	0	5	1	686
3709389	A	TRUE	G	100	0	0	24	0	G	100	0	0	11	0	G	100	6	5	547	5
3813389	G	TRUE	A	100	32	0	0	0	A	100	15	0	1	0	A	100	291	1	5	0
3915239	A	TRUE	C	100	0	8	0	0	C	91	0	3	0	0	C	100	4	164	1	0
3932468	G	TRUE	T	100	0	0	0	15	T	100	0	0	0	16	T	100	1	1	2	404
3992738	G	TRUE	A	100	30	0	0	0	A	100	36	0	0	0	A	100	212	0	1	0
4050459	C	TRUE	T	100	0	0	0	13	T	100	0	0	1	10	T	100	0	1	0	316
4052884	C	TRUE	T	100	0	0	0	24	T	100	0	0	0	24	T	100	0	8	2	418
4054898	C	TRUE	T	100	0	0	0	19	T	100	0	0	2	10	T	100	1	2	0	397
4058565	C	TRUE	T	100	0	0	0	16	T	100	0	0	0	30	T	100	0	7	0	139
4059099	C	TRUE	T	100	0	0	0	12	T	100	0	0	0	8	T	100	1	6	0	266
4061365	C	TRUE	T	100	0	0	0	22	T	100	0	0	0	10	T	100	3	0	0	471
4080297	T	TRUE	C	100	0	26	0	0	C	100	0	8	0	0	C	100	1	227	2	14
4082125	G	TRUE	A	100	11	0	0	0	A	100	10	1	0	0	A	100	377	16	5	3
4087153	C	TRUE	A	100	36	0	0	0	A	100	11	0	0	0	A	100	216	49	4	4
4116232	G	TRUE	A	100	19	0	0	0	A	100	5	0	0	0	A	100	356	6	5	3
4134023	A	TRUE	G	100	0	0	26	0	G	100	0	0	11	1	G	100	8	3	689	0
4154211	T	TRUE	G	100	0	0	19	0	G	100	0	0	29	0	G	100	1	3	174	18
4223342	C	TRUE	T	100	0	1	0	17	T	100	0	0	1	11	T	100	1	31	1	187
4226333	C	TRUE	T	100	0	0	0	29	T	100	0	0	1	17	T	100	0	4	0	522
4227228	T	TRUE	C	100	0	18	0	0	C	100	0	19	0	0	C	100	1	254	0	8
4238069	C	TRUE	T	100	0	0	0	18	T	100	0	0	2	12	T	100	0	12	5	428
4253274	C	TRUE	T	100	0	0	0	21	T	100	0	0	1	10	T	100	9	17	11	239
4269258	C	TRUE	T	100	0	0	0	27	T	100	1	0	1	16	T	100	1	2	13	474
4273039	C	TRUE	T	100	0	0	0	10	T	100	0	0	0	15	T	100	3	2	2	471
4274720	C	TRUE	T	100	0	0	0	24	T	100	0	0	0	5	T	100	1	12	1	235
4282078	C	TRUE	T	100	0	0	0	14	T	100	0	0	0	7	T	100	2	14	0	423
4286126	C	TRUE	T	100	0	0	0	17	T	100	0	0	1	22	T	100	0	1	0	195
4287667	C	TRUE	T	100	0	0	0	17	T	100	1	0	0	14	T	100	1	4	1	420
4289623	C	TRUE	T	100	0	0	0	22	T	100	0	0	0	4	T	100	1	16	1	224
4300948	C	TRUE	T	100	0	0	0	27	T	100	1	0	0	17	T	100	3	2	0	614
4307234	C	TRUE	T	100	0	0	0	23	T	100	0	0	0	7	T	100	1	17	4	330
4316015	C	TRUE	T	100	0	0	0	24	T	100	0	0	0	17	T	100	1	7	0	690
4324757	C	TRUE	T	100	0	0	0	21	T	100	0	0	1	8	T	100	6	19	6	101
4325463	C	TRUE	T	100	0	0	0	19	T	100	0	0	0	4	T	100	3	14	6	288
4330322	C	TRUE	T	100	0	0	0	21	T	100	0	0	0	16	T	100	1	5	1	170
4336988	C	TRUE	T	100	1	0	0	19	T	100	0	0	0	10	T	100	0	22	2	977
4342542	C	TRUE	T	100	0	0	0	21	T	100	0	1	2	40	T	100	2	1	0	334
4347503	C	TRUE	T	100	0	0	0	19	T	100	1	0	1	19	T	100	1	4	0	258
4353937	C	TRUE	T	100	0	0	0	20	T	100	0	0	0	13	T	100	0	2	0	134
4356197	C	TRUE	T	100	0	0	0	20	T	100	0	0	0	10	T	100	0	9	1	317
4356268	C	TRUE	T	100	0	0	0	24	T	100	0	0	1	44	T	100	1	2	1	257
4364587	C	TRUE	T	100	0	0	0	17	T	100	0	0	1	13	T	100	2	8	0	878
4369476	C	TRUE	T	100	0	0	0	31	T	100	0	0	0	16	T	100	0	3	2	267
4371348	C	TRUE	T	100	0	0	0	10	T	100	0	0	0	11	T	100	0	20	2	583
4376081	C	TRUE	T	100	0	0	0	30	T	100	0	0	2	17	T	100	0	7	1	696
4378101	C	TRUE	T	100	0	0	0	31	T	100	0	0	0	11	T	100	1	6	0	874
4385149	C	TRUE	T	100	0	0	0	15	T	100	0	0	3	19	T	100	2	33	1	363
4392800	C	TRUE	T	100	0	0	0	20	T	100	0	2	0	18	T	100	1	2	0	677
4394518	C	TRUE	T	100	0	0	0	26	T	100	0	0	0	12	T	100	0	9	0	602
4406287	C	TRUE	T	100	0	0	0	26	T	100	0	0	0	23	T	100	1	3	1	777
4407690	C	TRUE	T	100	0	0	0	31	T	100	0	0	0	11	T	100	1	14	1	349
4411725	C	TRUE	T	100	0	0	0	24	T	100	1	1	0	14	T	100	0	6	0	754
4419305	C	TRUE	T	100	0	0	0	27	T	100	0	0	1	8	T	100	1	16	0	156
4423542	C	TRUE	T	100	0	0	0	33	T	100	0	0	0	6	T	100	8	16	6	280
4431609	C	TRUE	T	100	0	0	0	33	T	100	0	0	1	24	T	100	1	16	1	695
4438139	C	TRUE	T	100	0	0	0	22	T	100	0	0	1	4	T	100	0	3	0	530
4439804	C	TRUE	T	100	0	0	0	20	T	100	0	0	0	33	T	100	1	13	1	531
4440430	C	TRUE	T	100	0	0	0	21	T	100	0	1	0	23	T	100	0	9	0	593
4455960	C	TRUE	T	100	0	0	0	22	T	100	0	0	0	13	T	100	0	5	0	641
4462072	C	TRUE	T	100	0	0	0	22	T	100	0	0	1	16	T	100	0	13	0	465
4487581	C	TRUE	T	100	0	0	0	25	T	100	0	0	0	20	T	100	1	3	0	208
4491549	T	TRUE	A	100	18	0	0	0	A	100	11	0	0	0	A	100	805	0	1	4
4491702	C	TRUE	T	100	0	0	0	29	T	100	0	0	0	28	T	100	1	6	1	948
4494455	C	TRUE	T	100	0	0	0	21	T	100	0	0	0	9	T	100	8	8	2	630
4499179	C	TRUE	T	100	0	0	0	9	T	100	0	0	1	35	T	100	0	7	0	655
4514918	T	TRUE	G	100	0	0	27	0	G	100	0	0	23	0	G	100	0	0	436	5
4522035	C	TRUE	T	100	0	0	0	18	T	100	0	0	0	8	T	100	0	3	2	492
4529081	C	TRUE	T	100	0	0	0	13	T	100	0	0	0	11	T	100	3	0	0	406
4529276	C	TRUE	T	100	0	0	0	30	T	100	0	0	1	16	T	100	5	44	3	552
4533719	C	TRUE	T	100	0	0	0	25	T	100	0	0	0	20	T	100	5	9	1	954
4535477	C	TRUE	T	100	0	0	0	26	T	100	0	0	0	11	T	100	1	6	1	480
4545941	C	TRUE	T	100	0	0	0	12	T	100	0	0	3	20	T	100	2	7	14	498
4569065	C	TRUE	T	100	0	0	0	16	T	100	0	0	0	11	T	100	1	5	0	476
4575230	C	TRUE	T	100	0	0	0	23	T	100	0	0	2	14	T	100	0	1	0	142
4575960	C	TRUE	T	100	0	0	0	18	T	100	2	0	0	21	T	100	2	11	4	639
4590353	C	TRUE	T	100	0	0	0	23	T	100	0	1	0	14	T	100	1	2	2	672
4592045	C	TRUE	T	100	0	0	0	17	T	100	1	0	3	19	T	100	40	8	0	318
4609197	C	TRUE	T	100	0	0	0	32	T	100	0	0	1	26	T	100	0	7	2	270
4634975	C	TRUE	T	100	0	0	0	28	T	100	0	0	0	6	T	100	2	13	1	634
4641605	G	TRUE	C	100	0	13	0	0	C	100	0	20	0	0	C	100	1	328	3	5
4642920	G	TRUE	A	100	16	0	0	0	A	100	12	0	0	5	A	100	1041	0	7	3
QV represents Phred score (i.e., -10 log10(p) ) of the consensus call in each method.
